# Supplementary material for: Indication of Strongly Correlated Electron Transport and Mott Insulator in Disordered Multilayer Ferritin Structures (DMFS)
Source: Materials (Basel). 2021 Aug 12;14(16):4527. doi: 10.3390/ma14164527 (PMC8399281; doi:10.3390/ma14164527)
Supplement: Supplementary file 1 [file materials-14-04527-s001.zip › materials-1305320-supplementary.pdf]

materials

MDPI

Supplementary Materials

Indication of Strongly Correlated Electron Transport and Mott Insulator in Disordered Multilayer Ferritin Structures (DMFS)

Christopher Rourk [1,*,†], Yunbo Huang [2,3,†], Minjing Chen [2] and Cai Shen [2,3,*]

[1] Independent Researcher, Dallas 75205, TX, USA
[2] Ningbo Institute of Materials Technology & Engineering Chinese Academy of Sciences. 1219 Zhongguan Road, Zhenhai District, Ningbo 315201, China; huangyunbo@nimte.ac.cn (Y.H.); chenminjing@nimte.ac.cn (M.C.)
[3] College of Materials Science and Opto-Electronic Technology, University of Chinese Academy of Sciences, Beijing 100049, China
* Correspondence: crourk@jw.com (C.R.); shencai@nimte.ac.cn (C.S.)
† equal contribution.

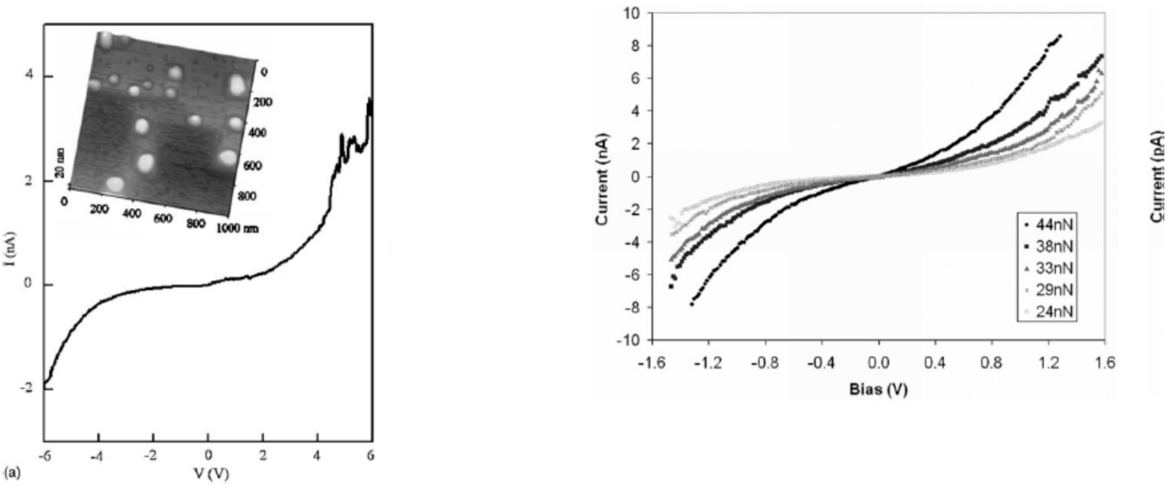

(A)	(B)

Figure S1. Comparison of semiconductor QD (from [1]) and ferritin I/V curves (from [2]).

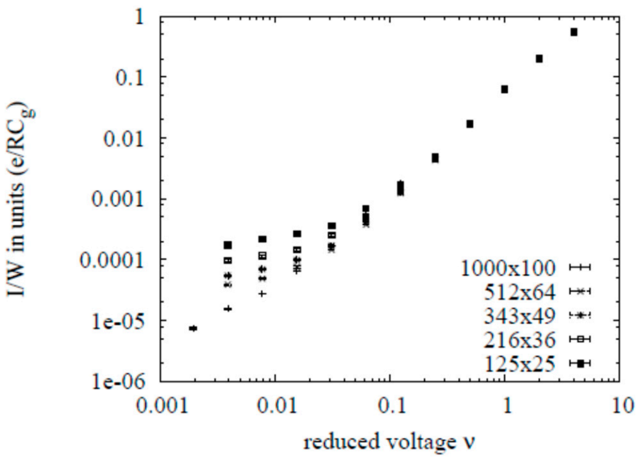

Figure S2. I/V characteristics through 2D arrays of QDs (from [6]).

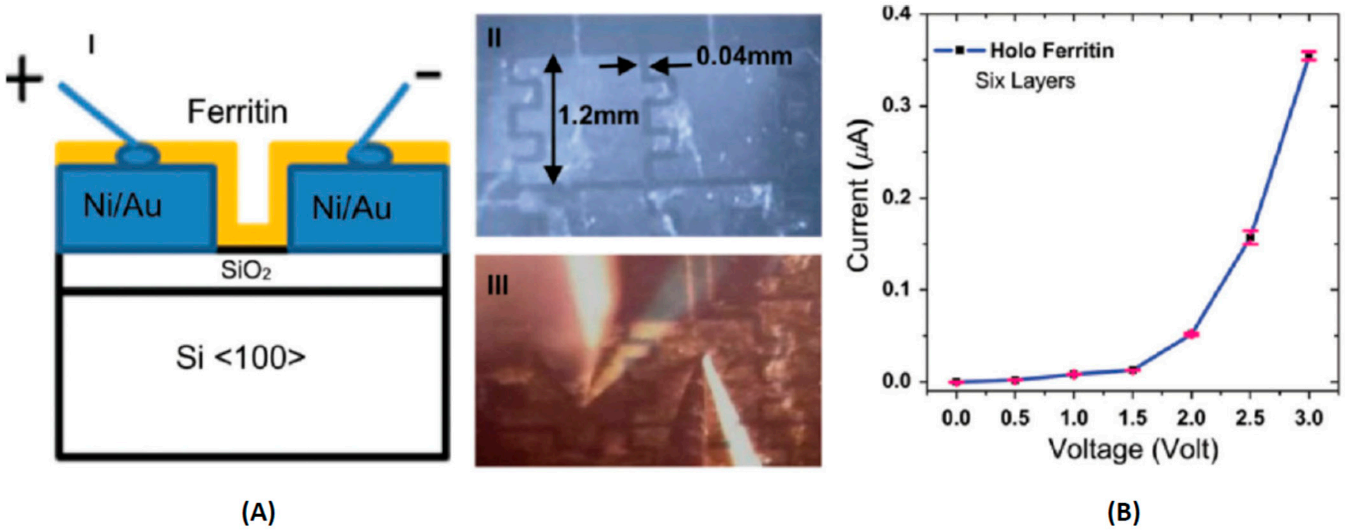

Figure S3. (**a**) circuit design and (**b**) current voltage behavior observed in [14].

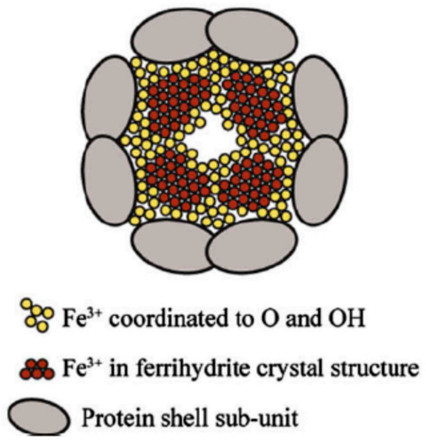

Figure S4. Ferritin structure, from [7].

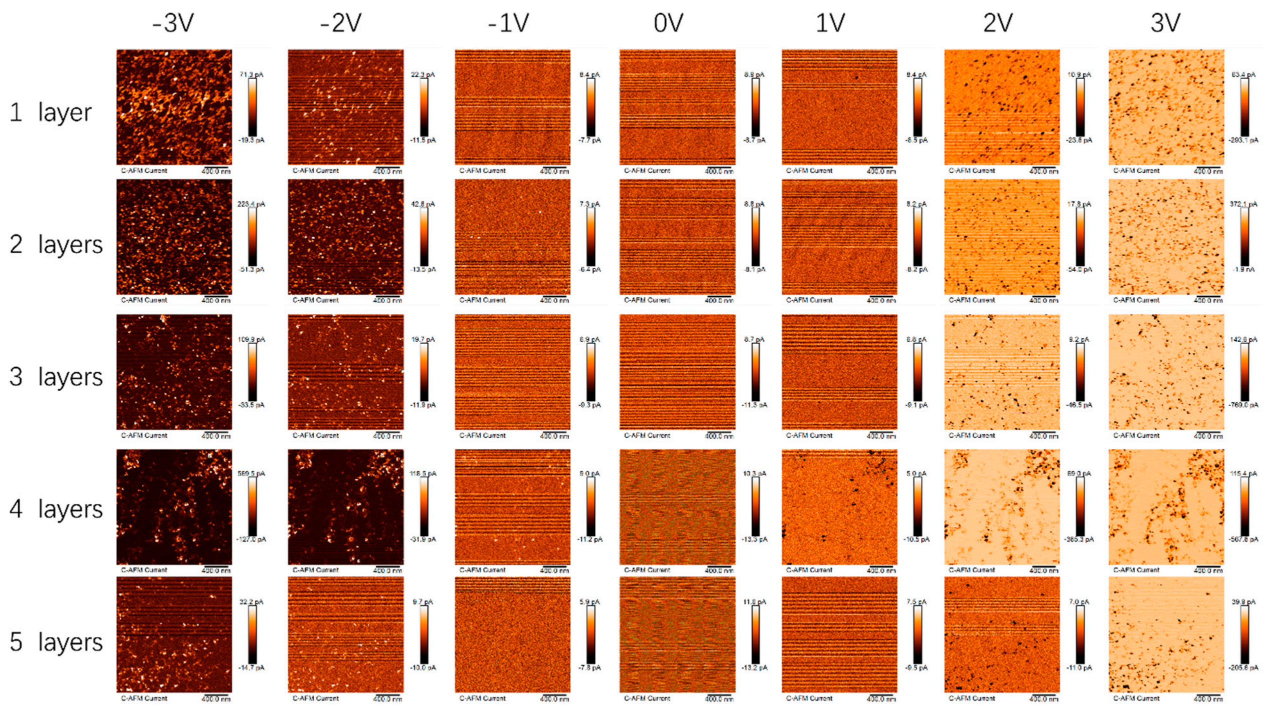

Figure S5. c-AFM image of ferritin on Si substrate.

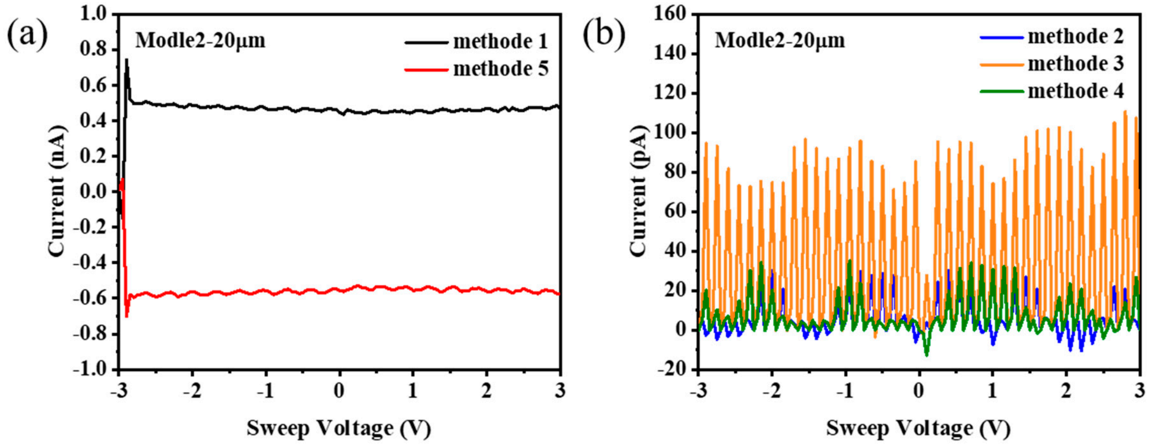

Figure S6. (**a**) I/V response between electrodes A1 and B1-B3 in parallel, no ferritin. (**b**) Electrodes B2 and B3 were left ungrounded, electrode B1 was grounded, and voltage was applied to electrode A1. .

Table S1. Summary of Results.

Layers of ferritin	Die type	Maximum current measured (20, 40 and 80 μm gap, respectively)
1	Model 1	noise, contaminated, damaged
1	Model 2	noise, noise, noise
2	Model 1	noise, noise, noise
2	Model 2	noise, 1 nA, noise
3	Model 1	--9 nA (retest --140 nA), 5 nA, damaged
3	Model 2	damaged, noise, noise
4	Model 1	---30 nA, --7 nA, damaged
4	Model 2	noise, noise, --3 μA
5	Model 1	noise, --3 μA, noise
5	Model 2	noise, noise, noise
6	Model 1	noise, noise, damaged
6	Model 2	damaged, noise, noise

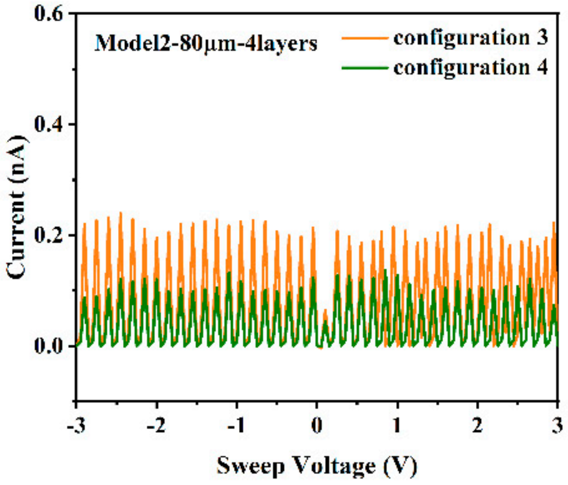

Figure S7. I/V curve for voltage applied to A1, B2 (left) and B3 (right) grounded,B1 and B3 float (left) and B1 and B2 float (right), 80 μm gap, 4 layers, model 2.

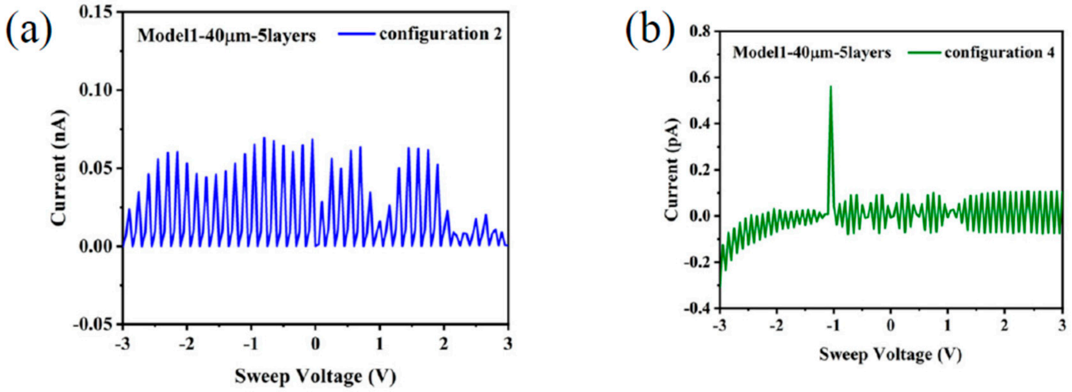

Figure S8. I/V curve for voltage applied to: (**a**) A1, B1 (left) and B3 (right) grounded, B2 and B3 float (configuration 2); and (**b**) B1 and B2 float (right), 40 μm gap, 5 layers (configuration 4).

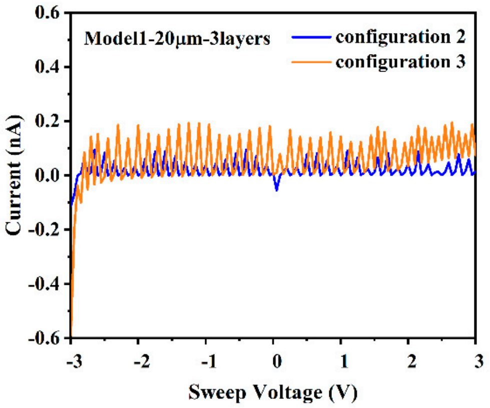

Figure S9. I/V curve for voltage applied to: a) A1, B1 (left) and B2 (right) grounded, B2 and B3 float (Configuration 2) and B1 and B3 float (Configuration 3), 20 μm gap, 3 layers.
